# Study of hsa_circRNA_000121 and hsa_circRNA_004183 in papillary thyroid microcarcinoma

**DOI:** 10.1515/biol-2022-0080

**Published:** 2022-07-12

**Authors:** Chao Bai, Wenwen Yang, Ru Ouyang, Zongbao Li, Li Zhang

**Affiliations:** Department of Vascular and Thyroid Surgery, The First Affiliated Hospital of Xinjiang Medical University, Urumqi 830054, China; The Second Department of General Internal Medicine, The First Affiliated Hospital of Xinjiang Medical University, Urumqi 830054, China; Department of Endocrinology, Sanya Central Hospital, No. 1154, Jiefang 4th Road, Tianya District, Sanya 572000, China

**Keywords:** PTMC, aggressiveness, hsa_circRNA_000121, hsa_circRNA_004183, SRC, MMP-14

## Abstract

We detected the expressions of hsa_circRNA_000121 and hsa_circRNA_ 004183 in papillary thyroid microcarcinoma (PTMC) and explored their relationship with the invasiveness of PTMC. PTMC patients with (*n* = 30; metastasis group) and without lymph node metastasis (*n* = 30; nonmetastasis group) were included. The levels of hsa_circRNA_000121, hsa_circRNA_004183, hsa-miR-4763, hsa-miR-6775, sarcoma gene (*SRC*), and *MMP-14* were detected with real-time polymerase chain reaction. Receiver-operating characteristic (ROC) analyzed the diagnostic value of hsa_circRNA_000121 and hsa_circRNA_004183. Binary logistic regression analysis evaluated the relationship of gene expression with PTMC invasiveness. In PTMC tissue samples, compared with the metastasis group, the expression of hsa_circRNA_000121, hsa_circRNA_004183, *SRC*, and *MMP-14* in the nonmetastasis group decreased, while the expression of hsa-miR-4763 and hsa-miR-6775 increased. In peripheral blood, compared with the metastasis group, the expression of hsa_circ_000121 and hsa_circRNA_004183 in the nonmetastasis group decreased. Both hsa_circRNA_000121 and hsa_circRNA_004183 had good sensitivity and specificity for diagnosing PTMC lymph node metastasis, with a cut-off value of 0.796 and 0.938, respectively. However, the gene expressions were not significantly associated with PTMC lymph node metastasis. Hsa_circRNA_000121 may upregulate *SRC* expression through hsa-miR-4763, while hsa_circRNA 000121 may upregulate MMP-14 expression through hsa-miR-6775, thereby promoting the aggressiveness of PTMC and ultimately leading to cervical lymph node metastasis. hsa_circRNA_000121 and hsa_circRNA_004183 may become potential biomarkers of PTMC aggressiveness.

## 1 Introduction

Thyroid cancer is the most common malignant tumor of the endocrine system and has the fastest-growing incidence in the world [1]. More than 50% of thyroid cancer is papillary thyroid microcarcinoma (PTMC) [2]. Most PTMC grows slowly and rarely develops into thyroid cancer of clinical significance. Thus, it has little effect on the survival rate of patients. A large clinical prospective study has verified the indolent growth pattern of PTMC [3]. However, not all PTMCs are “indolent” [4,5,6], and “small cancers with large metastases” are common [7]. A small number of PTMC patients have clinical manifestations like traditional papillary carcinoma, in which PTMC will continue to grow and show aggressiveness, such as cervical lymph node metastasis and postoperative recurrence, causing controversy over clinical treatment options [8]. A previous study showed that compared with patients with benign thyroid nodules and healthy subjects, the red blood cell distribution width, a new inflammatory marker, was increased in the blood of thyroid cancer patients, suggesting that it may be used as a marker of thyroid cancer [9]. Therefore, it is very important to identify the biological markers of PTMC invasiveness and explore the mechanism of PTMC invasiveness.

Circular RNA (circRNA) is a highly conserved noncoding RNA [10,11]. They are produced from pre-mRNAs by back-splicing [12]. They have tissue- and cell-specific expression patterns in eukaryotes. The biological functions of circRNA mainly include circRNA as microRNA (miRNA) sponge, circRNA–protein interaction, and encoding. circRNA may be involved in the occurrence and progression of the disease by regulating the expression of miRNA and protein [12]. The relationship between circRNA and malignant tumors has recently attracted much attention [13,14,15,16]. circRNA is more stable than linear RNA in the peripheral blood [17]. One study [18] reported that thousands of circRNAs were found in clinical whole blood samples. Peripheral blood circRNA detection has the characteristics of easy availability, high sensitivity, and convenience. It may be a potential ideal molecular marker for assessing tumor invasiveness. Previously, we used a gene chip to detect serum circRNA in PTMC patients with and without lymph node metastasis and obtained 690 differentially expressed circRNAs in serum, of which 400 were upregulated and 290 were downregulated [19]. The results suggest that hsa_circRNA_000121 may regulate PTMC invasiveness by inhibiting the effects of hsa-miR-4763 and hsa-miR-6775. However, the biological markers for the aggressiveness of PTMC are still lacking.

Herein, we aim to identify biomarkers related to PTMC invasiveness. PTMC patients without central lymph node metastasis were included in the nonmetastasis group, and PTMC patients with central lymph node metastasis were included in the metastasis group. We collected general clinical data, peripheral blood, and tissue specimens of all enrolled patients. Then, we performed real-time polymerase chain reaction (PCR) to detect expression levels of hsa_circRNA_000121 and hsa_circRNA_004183 in PTMC tissues and peripheral blood. Meanwhile, we detected the levels of hsa_circRNA_000121, hsa-miR-4763, hsa-miR-6775, sarcoma gene (*SRC*), and MMP-14 in PTMC tissues. The diagnostic value of hsa_circRNA_000121 and hsa_circRNA_004183 was assessed with the ROC analysis. The relationship of these genes with PTMC invasiveness was further evaluated. Our data may help understand the mechanism of PTMC invasiveness and may provide potential biological markers of PTMC invasiveness.

## 2 Materials and methods

### 2.1 Study subjects

We included PTMC patients with lymph node (central area) metastasis (*n* = 30; metastasis group) and those without lymph node (central area) metastasis (*n* = 30; nonmetastasis group) who were hospitalized in the Thyroid Surgery Department of the First Affiliated Hospital of Xinjiang Medical University from December 2019 to December 2021. Inclusion criteria were as follows: (1) patients with PTMC of lesion diameter less than 1 cm confirmed by surgery and pathology; (2) patients older than 18 years; and (3) patients received surgical treatment for the first time; Exclusion criteria were as follows: (1) patients with a clear history of thyroid disease; (2) patients with a history of thyroid surgery; (3) patients who had taken oral drugs for thyroid therapy before surgery; (4) pregnant women; (5) patients with abnormal liver and kidney function; and (6) patients with other malignant tumors. Peripheral blood was obtained from each patient. PTMC tumor tissues were collected from 15 patients of each group.


**Informed consent:** Informed consent has been obtained from all individuals included in this study.
**Ethical approval:** The research related to human use has been complied with all the relevant national regulations, institutional policies and in accordance with the tenets of the Helsinki Declaration, and has been approved by the ethics review board of the First Affiliated Hospital of Xinjiang Medical University (S20201106-01).

### 2.2 Real-time PCR

Total RNA was extracted from peripheral blood and PTMC tumor tissues, respectively, using Trizol reagent (#15596-026; Invitrogen, CA, USA) and then reverse transcribed into cDNA with a reverse transcription kit (#K1622; Thermo; San Jose, CA, USA). The levels of hsa_circRNA_000121 and hsa_circRNA_004183 in the peripheral blood and the expressions of hsa_circRNA_000121, hsa_circRNA_004183, hsa-miR-4763, hsa-miR-6775, *SRC*, and MMP-14 in PTMC tissues were tested by real-time PCR. The primers were synthesized by Sangon Biotech (Shanghai, China), and the primer sequences are presented in Table 1. Real-time PCR was conducted with SYBRGreen PCR kit (#F-415XL; Thermo, San Jose, CA, USA) on ABI-7500 (Applied Biosystems, Foster City, CA, USA). The real time-PCR reaction system (20 μL) included ddH_2_O 7.0 μL, SYBR Green qPCR Master Mix (2×) 10.0 μL, forward primer (10 μM) 1.0 μL, reverse primer/unified reverse primers (URP) (10 μM) 1.0 μL, and cDNA 1.0 μL. The PCR amplification conditions were 94°C for 10 min, and 40 cycles of 94°C for 20 s, 55°C for 20 s, and 72°C for 20 s. The 2^−△△CT^ method was used to calculate the relative expression of the genes in each group.

**Table 1:** Primers for real-time PCR

Primer	Sequence (5′–3′)
circRNA_000121-F	GACACCAGCCAGCCCTACA
circRNA_000121-R	ATTCTGCTCCCAATCCCTC
circRNA_004183-F	CCCCACTACGTCCATTCCA
circRNA_004183-R	AGCCTCTGACGCAGGGTTT
miRNA-6775-3p-F	ACACTCCAGCTGGGAGGCCCTGTCCTCTG
miRNA-4763-3p-F	ACACTCCAGCTGGGAGGCAGGGGCUGGUGCTG
Universal URP	TGGTGTCGTGGAGTCG
SRC (human)-F	AGCGGCGGCTTCTACATCAC
SRC (human)-R	GCCTCTGGAGACATCGTGCC
MMP14 (human)-F	TTGAGGTGGACGAGGAGGG
MMP14 (human)-R	GGGAACGCTGGCAGTAGAG
U6-F	CTCGCTTCGGCAGCACA
U6-R	AACGCTTCACGAATTTGCGT
GAPDH (human)-F	AGAAGGCTGGGGCTCATTTG
GAPDH (human)-R	AGGGGCCATCCACAGTCTTC

Note: SRC: sarcoma gene, MMP-14: matrix metalloproteinase 14, GAPDH: glyceraldehyde-3-phosphate dehydrogenase.

### 2.3 Statistical analysis

All data are analyzed by SPSS20.0 software (IBM, Chicago, IL, USA). The measurement data with normal distribution are expressed as mean ± SD. Then, *t* test was used for comparison between groups. The measurement data with nonnormal distribution are represented by the median and interquartile range and compared with the rank-sum test. Enumeration data are expressed as the absolute number of cases and percentages and were analyzed with *χ* ^2^ test. ROC was used to evaluate the diagnostic value of hsa_circ_000121 and hsa_circRNA_004183. Binary logistic regression analysis was used to evaluate the relationship between the expression levels of hsa_circRNA_000121 and hsa_circRNA_004183 in the peripheral blood and the expression levels of hsa_circRNA_000121, hsa-miR-4763, hsa-miR-6775, *SRC*, and MMP-14 in PTMC tissues with PTMC invasiveness. *P* < 0.05 indicates that the difference is statistically significant.

## 3 Results

### 3.1 Clinical data of included subjects

In this study, the peripheral blood was obtained from 30 cases of PTMC patients with metastasis (metastasis group) and 30 cases of PTMC patients without metastasis (nonmetastasis group). The average age of patients in the metastasis group was 41.07 ± 11.67 years, and the average age of the nonmetastasis group was 45.10 ± 8.54 years (*t* = 1.528, *P* = 0.132). The metastasis group included 13 males (43.33%) and 17 females (56.67%). The nonmetastasis group included 6 males (20%) and 24 females (80%). There was no significant difference in the sex ratio between the two groups (*χ* ^2^ = 3.774, *P* = 0.052).

In addition, PTMC tumor tissues were collected from another 15 cases of PTMC patients with metastasis (metastasis group) and 15 cases of PTMC patients without metastasis (nonmetastasis group). For their clinical data, the average age of the metastasis group was 46.40 ± 9.72 years and that of the nonmetastasis group was 46.13 ± 9.74 years (*t* = 0.075, *P* = 0.941). There were five males (33.33%) and 10 females (66.67%) in the metastasis group and seven males (46.67%) and eight females (53.33%) in the nonmetastasis group (*χ* ^2^ = 0.556, *P* = 0.456).

### 3.2 Detection of circRNA levels by real-time PCR

**Figure 1:**
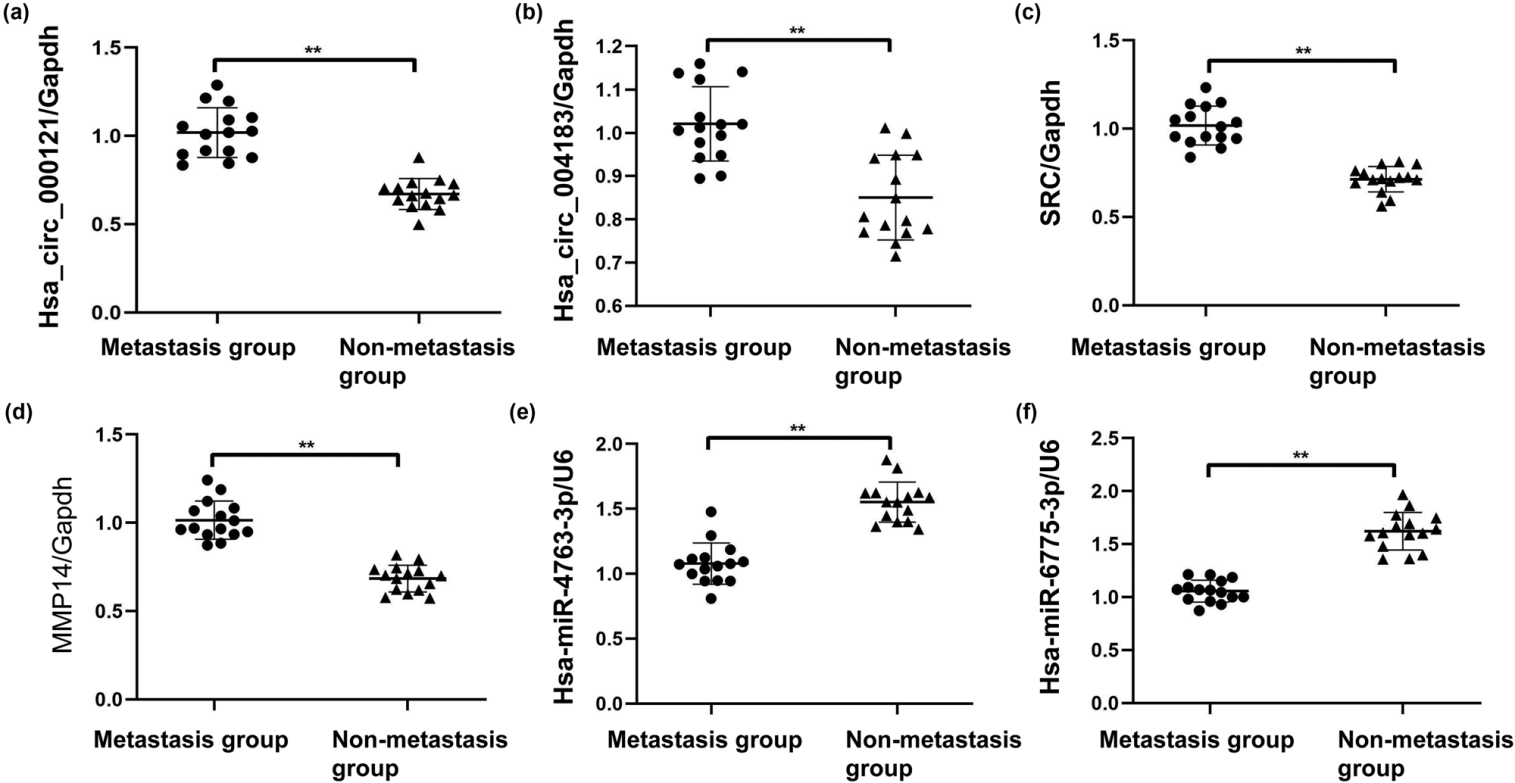
Differences in gene expression between nonmetastasis patients and those with metastasis in tissues. Gene expression in tissues was detected with real-time PCR. (a) Comparison of hsa_circRNA_000121 expression in tissues. (b) Comparison of hsa_circRNA_004183 expression in tissues. (c) Comparison of SRC expression levels in tissues. (d) Comparison of MMP-14 expression levels in tissues. (e) Comparison of expression levels of hsa-miR-4763 in tissues. (f) Comparison of hsa-miR-6775 expression levels in tissues.

In PTMC tumor tissues, compared with the nonmetastasis group, the expression of hsa_circRNA_000121 (Figure 1a), hsa_circRNA_004183 (Figure 1b), *SRC* (Figure 1c), and MMP-14 (Figure 1d) increased significantly in the metastasis group (*P* < 0.05). However, the expression of hsa-miR-4763 (Figure 1e) and hsa-miR-6775 (Figure 1f) decreased significantly in the metastasis group (*P* < 0.05; Table 2).

**Table 2:** Comparison of levels of circRNA, miRNA, and mRNA in tumor tissues

Group	hsa_circRNA_000121	hsa_circRNA_004183	hsa-miR-4763	hsa-miR-6775	SRC	MMP−14
Metastasis group	0.671 ± 0.876	0.850 ± 0.979	1.552 ± 0.154	1.622 ± 0.176	0.714 ± 0.719	0.684 ± 0.076
Nonmetastasis group	1.019 ± 0.141	1.021 ± 0.858	1.078 ± 0.159	1.056 ± 0.103	1.019 ± 0.110	1.015 ± 0.108
*t*	8.137	5.068	−8.275	−10.734	8.984	9.730
*P*	<0.01	<0.01	<0.01	<0.01	<0.01	<0.01

Note: SRC: sarcoma gene, MMP-14: matrix metalloproteinase 14.

**Figure 2:**
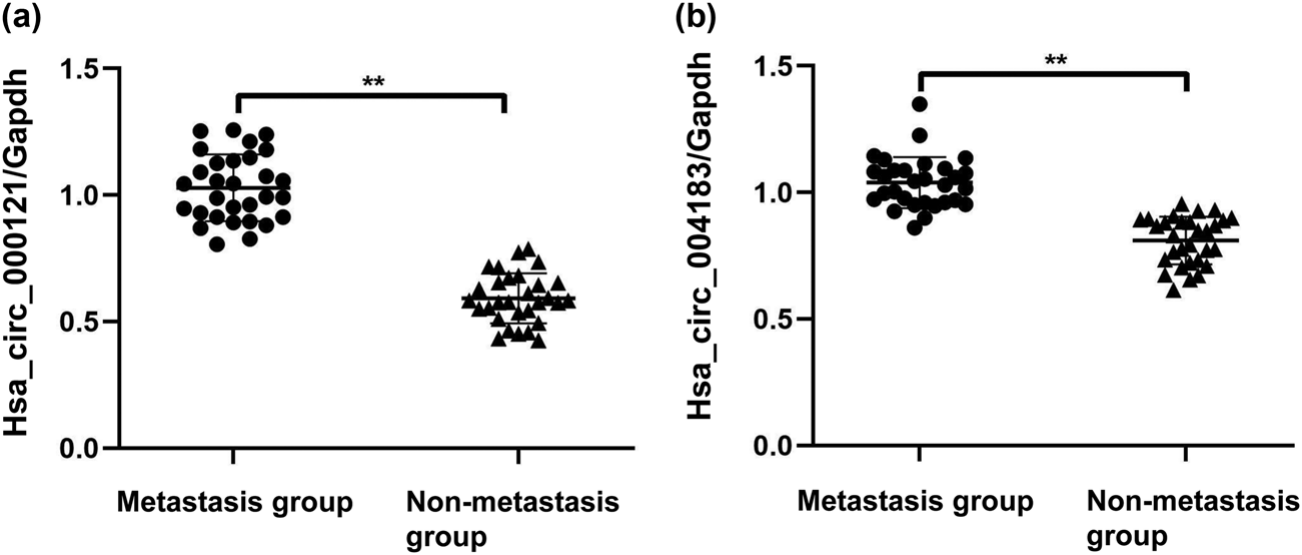
Differences in gene expression between nonmetastasis patients and those with metastasis in peripheral blood. Gene expression in the peripheral blood was detected with real-time PCR. (a) Comparison of hsa_circRNA_000121 expression in the peripheral blood. (b) Comparison of hsa_circRNA_004183 expression in the peripheral blood.

In the peripheral blood, compared with the nonmetastasis group, the expression of hsa_circRNA_000121 (Figure 2a) and hsa_circRNA_004183 (Figure 2b) significantly increased in the metastasis group (*P* < 0.05; Table 3), which was consistent with their expression in PTMC tumor tissues.

**Table 3:** Comparison of circRNA levels in peripheral blood

Group	hsa_circRNA_000121	hsa_circRNA_004183
Metastasis group	0.592 ± 0.986	0.810 ± 0.946
Nonmetastasis group	1.029 ± 0.132	1.039 ± 0.101
*t*	−14.553	−9.055
*P*	<0.01	<0.01

### 3.3 The hsa_circRNA_000121 and hsa_circRNA_004183 have good diagnostic values for PTMC lymph node metastasis

**Figure 3:**
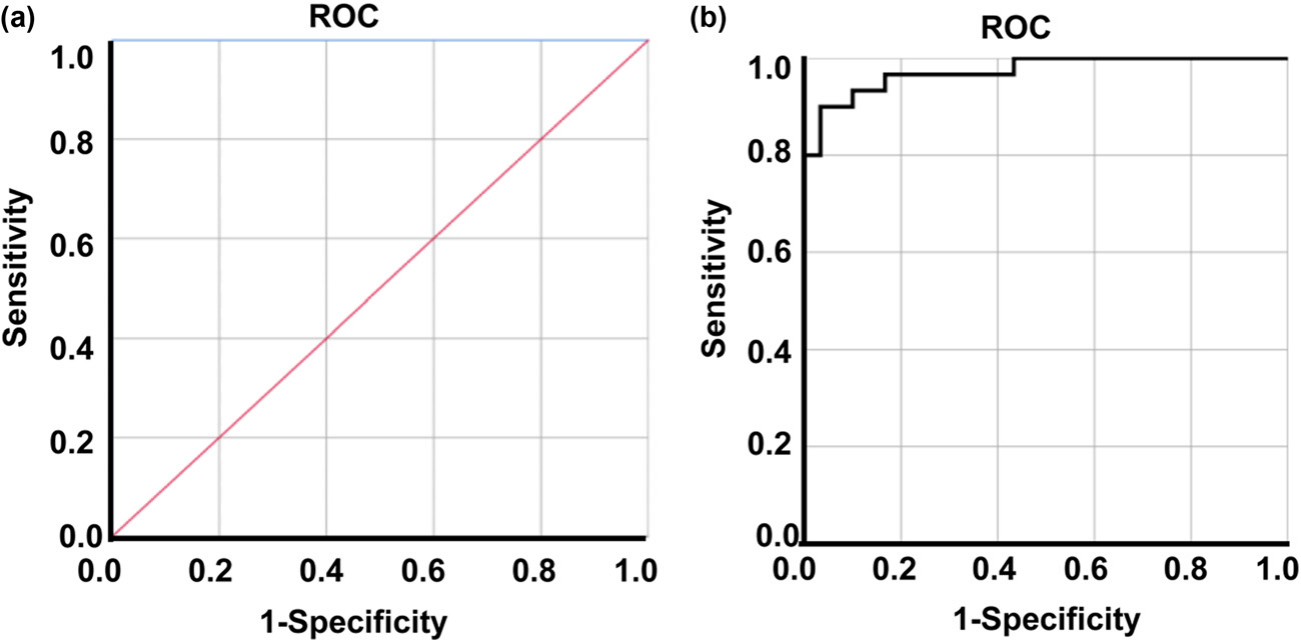
ROC analysis of hsa_circRNA_000121 and hsa_circRNA_004183. (a) ROC curve of hsa_circRNA_000121. (b) ROC curve of hsa_circRNA_004183.

Next, to evaluate the diagnostic value of hsa_circ_000121 and hsa_circRNA_004183, ROC analysis was performed. For hsa_circRNA_000121, the area under the curve (AUC) was 1.000, the cut-off value (Youden index) was 0.796, the sensitivity was 100%, and the specificity was 100% (Figure 3a). In addition, the AUC of hsa_circRNA_004183 was 0.973, with a cut-off value (Youden index) of 0.939, the sensitivity of 90%, and the specificity of 96.7% (Figure 3b). These results indicate that both hsa_circ_000121 and hsa_circRNA_004183 have good sensitivity and specificity for diagnosing PTMC.

### 3.4 The expression of circRNAs has no correlation with PTMC lymph node metastasis

Binary logistic regression analysis showed that the expression levels of circRNA-000121 and hsa_circRNA_004183 in the peripheral blood (Table 4) as well as the expression levels of hsa_circRNA_000121, hsa_circRNA_004183, hsa-miR-4763, hsa-miR-6775, *SRC*, and MMP-14 in PTMC tumor tissues (Table 5) were not associated with PTMC lymph node metastasis.

**Table 4:** Binary Logistic regression analysis of the expression levels of circRNA-000121 and hsa-miR-4763 in peripheral blood

Variables	β	SD	Wald value	Freedom	*P*
hsa_circRNA_000121	47.681	67571.054	0.000	1	0.999
hsa_circRNA_004183	11.519	75445.917	0.000	1	1.000
Constant	−66.739	75308.807	0.000	1	0.999

**Table 5:** Binary logistic regression analysis of the expression levels of circRNA-000121, hsa-miR-4763, and SRC in PTMC tumor tissues

Variables	*β*	SD	Wald value	Freedom	*P*
hsa_circRNA_000121	−17.572	113497.280	0.000	1	1.000
hsa-miRNA-4763	37.046	142909.222	0.000	1	1.000
SRC	−66.128	353776.182	0.000	1	1.000
hsa_circRNA_004183	−49.258	202822.534	0.000	1	1.000
hsa-miRNA-6775	22.292	136076.097	0.000	1	1.000
MMP-14	−11.169	166352.188	0.000	1	1.000
Constant	45.844	133480.481	0.000	1	1.000

Note: SRC: sarcoma gene, MMP-14: matrix metalloproteinase 14.

## 4 Discussion

circRNAs are rich in miRNA response elements and act as miRNA sponges in cells [20]. They can act as competitive endogenous RNAs and combine with miRNA to release the inhibition of miRNA on its target genes and restore the expression level of target genes [21]. Therefore, circRNAs may regulate the occurrence and development of tumors by regulating signal transduction, may serve as potential biomarkers [22,23], and may regulate the expression of target genes as miRNA sponges [24,25,26,27].

miRNA is a kind of noncoding miRNA with a length of 21−23 nt. It can downregulate gene expression by causing mRNA degradation or inhibiting protein translation, thereby participating in regulating the development, proliferation, and differentiation of tumor cells [28,29]. Some miRNAs can upregulate tumor suppressor gene expression and inhibit tumor occurrence and development, while some miRNAs can exert tumor-promoting effects and play a role in inducing malignant transformation of cells [30]. It is suggested that the expression of miR-4763 was downregulated in multidrug-resistant gastric cancer cells, indicating that miR-4763 may be related to the drug resistance of gastric cancer [31]. Another study [32] found that hsa-miR-4763-3p was significantly upregulated in lymphoma tissues. In addition, hsa-miR-4763-3p was reported to be upregulated in serum samples of patients with esophageal squamous cell carcinoma [33]. The aforementioned studies all suggest that miR-4763 may be involved in the occurrence and development of tumors. The results of this study revealed that the expression of hsa-miR-4763, which regulates *SRC*, was reduced in PTMC, suggesting that miRNA-4763 may play a role in the aggressiveness of PTMC via regulating the expression of *SRC*.

It has been [34] shown that miR-6775-3p can negatively regulate CDK4, CDK6, MMP17, and MMP24 levels in breast cancer cells by binding to the 3′UTR of their mRNAs, thereby inhibiting the proliferation, migration, and invasive abilities of breast cancer cells. Our results found that the expression of hsa-miR-6775 was decreased in PTMC with lymph node metastasis, indicating that hsa_circRNA_000121 may increase the expression level of MMP-14 by downregulating the expression of hsa-miR-6775, which may play a role in the invasion of PTMC.

*SRC* is a member of the SRC family of tyrosine kinases and is closely related to the occurrence and development of tumors. As one of the earliest identified oncogenes, *SRC* is involved in cell proliferation, adhesion, migration, apoptosis, and angiogenesis, as well as cell invasion and metastasis of malignant tumors [35]. By interacting with the epidermal growth factor receptor (EGFR), platelet-derived growth factor receptor, hepatocyte growth factor receptor, and G protein-coupled receptor, the downstream signaling pathway of SRC is activated [36,37]. SRC can promote epidermal growth factor-induced nonadherent growth and tumorigenesis in nude mice [38]. SRC is an effector of the EGFR signaling pathway, which enhances the invasiveness and survival rate of tumor cells [39]. In mouse models of glioma, selective inhibition of SRC affected the motility of EGFR-dependent tumor cells [40]. In addition, the combination of SRC inhibitors and EGFR blocking antibodies significantly inhibited tumor growth and prolonged the survival rate of mice [40]. The combination of SRC inhibitors and antitumor drugs could reverse the drug resistance of tumors and inhibit tumor metastasis and recurrence [41]. Compared with promoting tumor generation, SRC plays a more important role in the process of tumor invasion [42]. Current SRC inhibitors play an antitumor effect mainly by inhibiting tumor metastasis rather than tumor growth [43]. In this study, we collected samples from 30 PTMC patients without and with lymph node metastasis and compared the expression levels of hsa_circRNA_000121 and hsa-miR-4763 and SRC. The expression levels of hsa_circ_000121 and SRC in the metastasis group were significantly upregulated compared with the nonmetastasis group, while the expression levels of hsa-miR-4736 decreased. It is considered that hsa_circRNA_000121 may upregulate the expression level of SRC by combining with hsa-miR-4763, thereby enhancing the aggressiveness of PTMC and eventually leading to cervical lymph node metastasis. However, further experimental validation is needed.

MMP is a structurally zinc-dependent polypeptide endonuclease that hydrolyzes extracellular matrix protein components [44]. The MMP family member MMP14 has the functions of regulating cell growth [45], tumor invasion and metastasis [46], and key gene expression [47]. The plasma membrane anchoring domain is a distinctive feature of MMP-14 [48]. Yao et al. found that MMP-14 activated MMP2 to promote tumor invasion [49]. MMP-14 is highly expressed in hepatocellular carcinoma, pancreatic cancer, non-small-cell lung cancer, gastric cancer, and cervical cancer, among else, and is closely related to tumor size, type of invasion, pathological type, degree of differentiation, and metastasis [50,51,52,53]. The main mechanisms by which MMP-14 promotes the spread and growth of tumor cells are as follows: (1) It activates the c-Jun N-terminal kinase signaling pathway, increases the expression of MMP-9, and promotes tumor invasion [54]. (2) It binds to the hyaluronic acid receptor, promotes the contraction of actin, and affects tumor invasion [55]. (3) It promotes the expression level of vascular endothelial growth factors and other factors, inhibits the spread of semaphorin 4D, and promotes the formation of new tumor blood vessels [56]. (4) It decomposes extracellular matrix components and promotes tumor invasion [50]. Herein, we detected the expression of hsa_circRNA_000121, hsa-miR-6775, and MMP-14 in the PTMC tissues of the two groups of patients. The results showed that the expression of hsa_circ_000121 and MMP-14 in the metastasis group increased significantly compared with the nonmetastasis group, while the expression of hsa-miR-6775 decreased. Therefore, hsa_circRNA_000121 may upregulate the expression of MMP-14 by combining hsa-miR-6775, enhance the aggressiveness of PTMC, and ultimately lead to cervical lymph node metastasis in patients. However, further experimental validation is needed.

In addition, we found that expressions of hsa_circRNA_000121 and hsa_circRNA_004183 in the peripheral blood of PTMC patients with lymph node metastasis in the central area of the neck were significantly higher than those of PTMC patients without cervical lymph node metastasis. ROC analysis showed that the cut-off values of hsa_circRNA_000121 and hsa_circRNA_004183 in PTMC patients with cervical lymph node metastasis were 0.796 and 0.938, respectively. Thus, when the expression of hsa_circRNA_000121 in the peripheral blood of PTMC patients is higher than 0.796 or the hsa_circRNA_004183 expression in peripheral blood of PTMC patients is higher than 0.938, it is considered that there may be cervical lymph node metastasis.

Binary logistic regression analysis showed that the expressions of circRNA-000121, hsa-miR-6775, hsa-miR-4763, MMP-14, and SRC in PTMC tissues were not significantly related to lymph node metastasis. The expression of hsa_circRNA_000121 and hsa_circRNA_004183 in the peripheral blood was also not significantly related to lymph node metastasis. These results may be related to deficiencies in the experimental design. Due to the nonrigorous diagnostic test in this study, the included patients were not selected at random, resulting in greater selection bias.

## 5 Conclusion

In this study, we analyzed the expression of circRNA-000121, hsa-miR-6775, hsa-miR-4763, MMP-14, and SRC in PTMC tumor tissues and compared the levels of hsa_ircRNA_000121 and hsa_circRNA_004183 in the peripheral blood of patients. The results showed that in the peripheral blood, the expression levels of hsa_circ_000121 and hsa_circRNA_004183 in the nonmetastasis group were lower than those in the metastasis group. Besides, the expression of hsa_circ_000121 greater than 0.80 or hsa_circRNA_004183 greater than 0.94 in the peripheral blood suggests that PTMC may be more aggressive. These results imply that hsa_circ_0000121 and hsa_circRNA_004183 might become potential biomarkers of PTMC aggressiveness. However, further experimental validation is needed.
